# Alignment-free phylogeny of whole genomes using underlying subwords

**DOI:** 10.1186/1748-7188-7-34

**Published:** 2012-12-06

**Authors:** Matteo Comin, Davide Verzotto

**Affiliations:** 1 Department of Information Engineering, University of Padova, Padova, Italy

**Keywords:** Phylogeny, Alignment-free algorithms, Pattern discovery

## Abstract

**Background:**

With the progress of modern sequencing technologies a large number of complete genomes are now available. Traditionally the comparison of two related genomes is carried out by sequence alignment. There are cases where these techniques cannot be applied, for example if two genomes do not share the same set of genes, or if they are not alignable to each other due to low sequence similarity, rearrangements and inversions, or more specifically to their lengths when the organisms belong to different species. For these cases the comparison of complete genomes can be carried out only with ad hoc methods that are usually called alignment-free methods.

**Methods:**

In this paper we propose a distance function based on subword compositions called Underlying Approach (UA). We prove that the matching statistics, a popular concept in the field of string algorithms able to capture the statistics of common words between two sequences, can be derived from a small set of “independent” subwords, namely the irredundant common subwords. We define a distance-like measure based on these subwords, such that each region of genomes contributes only once, thus avoiding to count shared subwords a multiple number of times. In a nutshell, this filter discards subwords occurring in regions covered by other more significant subwords.

**Results:**

The Underlying Approach (UA) builds a scoring function based on this set of patterns, called underlying. We prove that this set is by construction linear in the size of input, without overlaps, and can be efficiently constructed. Results show the validity of our method in the reconstruction of phylogenetic trees, where the Underlying Approach outperforms the current state of the art methods. Moreover, we show that the accuracy of UA is achieved with a very small number of subwords, which in some cases carry meaningful biological information.

**Availability:**

http://www.dei.unipd.it/∼ciompin/main/underlying.html

## Background

The global spread of low-cost high-throughput sequencing technologies has made a large number of complete genomes publicly available, and this number is still growing rapidly. In contrast, only few computational methods can really handle as input entire chromosomes, or entire genomes.

Traditionally the comparison of related genomes is carried out by sequence alignment. Popular methods extract gene-specific sequences from all species under examination and build a multiple sequence alignment for each gene [1]. Then all multiple sequence alignments are merged to form the final phylogeny. Other methods [2] use genes as a dictionary, counting the presence or absence of a gene. This gene profile is then used to derive a similarity score. However, if the genomes in question do not share a common set of genes, or if they cannot be aligned to each other, e.g., due to substantially different lengths, these traditional techniques cannot be applied. As a general example, in a pairwise comparison of genomes popular alignment tools rely on a specific order of elements for each genome sequence, and on a set of sparse shared seeds that should then be extended to obtain a global alignment. Therefore low sequence similarity, rearrangements, and inversions can cause major problems in identifying a possible alignment and thus the actual sequence similarity.

Furthermore, when considering whole genomes, the global alignment of large sequences has become a prohibitive task even for supercomputers, hence simply infeasible. To overcome these obstacles, in the last ten years a variety of alignment-free methods have been proposed. In principle they are all based on counting procedures that characterize a sequence based on its constituents, e.g., *k*-mers [3,4].

An important aspect in phylogeny reconstruction is the fact that gene-based methods strictly focus on comparing the coding regions of genomes, which can account for as little as 1% of the genomic sequence in humans [5]. Whereas it is known that the use of whole genomes may provide more robust information when comparing different organisms [6]. Also most alignment-free methods in the literature use only a portion of complete genomes [7]. For instance, there are approaches that use only genic regions [3] or mitochondria; other methods filter out regions that are highly repetitive or with low complexity [4]. Recently, it has been shown that the evolutionary information is also carried by non-genic regions [8]. For certain viruses, we are not even able to estimate a complete phylogeny by analyzing their genes, since these organisms may share a very limited genetic material [7].

Sims *et al.* recently applied the Feature Frequency Profiles method (FFP) presented in [4] to compute a whole-genome phylogeny of mammals [8]—i.e., large eukaryotic genomes including the human genome — and of bacteria. This method needs to estimate the parameter *k* in order to compute a feature vector for each sequence, where the vector represents the frequency of each possible *k*-mer. Each feature vector is then normalized by the total number of *k*-mers found (i.e., by the sequence length minus *k-1*), obtaining a probability distribution vector, or feature frequency profile, for each genome. FFP finally computes the distance matrix between all pairs of genomes by applying the Jensen-Shannon [9] divergence to their frequency profiles.

This general characterization of strings based on their subsequence composition closely resembles some of the information theory problems, and is tightly related with the compression of strings. In fact, compositional methods can be viewed as the reinterpretation of data compression methods, well known in the literature [10,11]. For a comprehensive survey on the importance and implications of data compression methods in computational biology, we refer the reader to [12].

When comparing entire genomes we want to avoid that large non-coding regions, which by nature tend to be highly repetitive, may contribute to our scoring function a multiple number of times, thus misleading the final similarity score. In fact, while analyzing massive genomes, the number of repeated patterns is very high, particularly in the non-genic regions. Furthermore if we allow mismatches the number of patterns can grows exponentially [13-15]. In this paper we will address this problem by controlling the distribution of subwords over the sequences under consideration, so that their contribution will not be overcounted.

Moreover, when comparing genomes it is well known that different evolutionary mechanisms can take place. In this framework, two closely related species are expected to share larger portions of DNA than two distant ones, whereby also other large complements and reverse-complements, or inversions, may occur [16]. In this work we will take into account all these symmetries, in order to define a measure of similarity between whole genomes.

### Matching statistics

Among the many distance measures proposed in the literature, which in most cases are dealing with *k*-mers, an effective and particularly elegant method is the Average Common Subword approach (ACS), introduced by Ulitsky *et al.*[7]. They use a popular concept in the field of string algorithms, known as matching statistics [17]. In short, given two sequences *s*_1_ and *s*_2_, where *s*_1_ is the reference sequence, the matching statistic is a vector *l* such that *l* [*i*] is the length of the longest subword starting at position *i* of *s*_1_ that is also a subword of *s*_2_, for every possible position *i* of *s*_1_(see Table 1).

**Table 1:** **Example of matching statistics *l***_**1**_**[*i*] and *l***_**2**_**[*j*] for the strings *s***_**1**_** = **ACACGTAC**and *s***_**2**_** = **TACGTGTA


*s*_1_[*i*]	A	C	A	C	G	T	A	C
*l*_1_[*i*]	2	1	4	3	3	3	2	1
*s*_2_[*j*]	T	A	C	G	T	G	T	A
*l*_2_[*j*]	3	4	3	2	1	3	2	1

A popular measure of similarity between strings is the average of this vector. In fact the general form of ACS is:

$$\text{ACS}(s_{1},s_{2})=\frac{\sum_{i=1}^{\left|s_{1}\right|}l[i]}{\left|s_{1}\right|}.$$

We can notice the similarity with the cross entropy of two probability distributions *P* and *Q*:

$$H(P,Q)=-\underset{x}{\sum }p(x)\text{log}q(x),$$

where *p*(*x*) log* q*(*x*) measures the number of bits needed to code an event *x* from *P* if a different coding scheme based on *Q* is used, averaged over all possible events *x*.

From the theoretical prospective it can be shown [7] that the ACS approach mimics the cross entropy estimated between two large sequences generated by a finite-state Markov process. In practice, this is closely related to the Kullback-Leibler information divergence, and represents the minimum number of bits needed to describe one string, given the other: *D*_*KL*_(*P *∥* Q*) =* H*(*P*,*Q*) −* H*(*P*). This is perhaps the most frequently used information-theoretic similarity measure.

The advantage of using the matching statistics is that it is not based on fixed-length subwords, but it can capture also variable length matches, in contrast to most methods that are based on fixed sets of *k*-mers. In fact, with the latter the choice of the parameter *k* is critical, and every method needs to estimate *k* from the data under examination, typically using empirical measurements [4].

For this reason ACS proved to be useful for reconstructing whole-genome phylogenies of viruses, bacteria, and eukaryotes, outperforming in most cases the state-of-the-art methods [7].

## Methods

In this section we propose a distance measure between entire genomes based on the notion of underlying subwords. In order to build a sound similarity measure between genomes, we need first to study the properties of the matching statistics. Our first contribution is the characterization of the subwords that are needed to compute the matching statistics. A second contribution is the selection of these subwords so that the resulting similarity measure does not contain overcounts. Our main idea is to avoid overlaps between selected subwords, more precisely by discarding common subwords occurring in regions covered by other more significant subwords.

### Irredundant common subwords

In the literature, the values *l*[*i*] used by the ACS approach are called the *matching statistics*, as described in detail by Gusfield [17]. Our first contribution is to characterize the matching statistics in order to identify which subwords are essentials.

It is well known that the total number of distinct subwords of any length found in a sequence of length *n* can be at most Θ(*n*^2^). Remarkably a notable family of fewer than *2n* subwords exist that is maximal in the host sequence, in the sense that it is impossible to extend a word in this class by appending one or more characters to it without losing some of its occurrences [18]. It has been shown that the matching statistics can be derived from this set of maximal subwords [19]. Here we will further tighten this bound by showing that to compute the matching statistics it is enough to consider a subset of the maximal subwords, called *irredundant common subwords*.

The notion of irredundancy was introduced in [20] and later modified for the problem of protein comparison [21,22]. It proved useful in different contexts from data compression [23] to the identification of transcription factors [24]. In this paper we introduce the concept of *irredundant common subwords* (i.e., without mismatches/wildcards). This ensures that there exists a close correspondence between the irredundant common subwords and the matching statistics.

#### Definition 1

(Irredundant/Redundant common subword) A common subword *w* is *irredundant* if and only if at least an occurrence of *w* in *s*_1_ or *s*_2_ is not covered by other common subwords. A common subword that does not satisfy this condition is called a redundant common subword.

We observe that the number of irredundant common subwords $I_{s_{1},s_{2}}$ is bounded by *m* + *n*, where |*s*_1_| =* n *and |*s*_2_| =* m*, since it is a subset of the set of *maximal common subwords* (see [19,25] for a more complete treatment of this topic).

#### Proposition 1

The matching statistics $l_{s_{1}}(i)$ can be computed by combining together all and only the irredundant common subwords of *s*_1_ and *s*_2_.

#### Proof

To show that the vector $l_{s_{1}}(i)$ can be derived from the irredundant common subwords, we define a new vector of scores *l*_*w *_for each subword *w*, where *l*_*w*_[*j*] = |*w*|−*j* + 1 represents the length of each suffix *j* of *w*, with *j *= 1,…,|*w*|. Then, for each subword *w* in $I_{s_{1},s_{2}}$ we superimpose the vector *l*_*w *_on all the occurrences of *w* in *s*_1_. For each position *i*, in *s*_1_, $l_{s_{1}}(i)$ is the maximum value of the scores *max*_*w*_(*l*_*w*_[*j*]), such that *k* + *j *=* i *and *k* is an occurrence of *w*.

To complete the proof we have to show that every occurrence of a common subword of *s*_1_ and *s*_2_ is covered by some occurrence of a subword in $I_{s_{1},s_{2}}$. By definition of irredundant common subword, any occurrence of a subword corresponds to an irredundant common subwords or is covered by some subword in $I_{s_{1},s_{2}}$. Moreover every irredundant common subword *w* has at least an occurrence *i* that is not covered by other subwords. Thus, $l_{s_{1}}(i)$ corresponds exactly to |*w*| and the subword *w* is necessary to compute the matching statistics. In conclusion, by using the method described above for $l_{s_{1}}(i)$, we can compute for each position the length of the maximum common subword starting in that location, which corresponds to the matching statistics. □

In summary, the notion of irredundant common subwords is useful to decompose the information provided by the matching statistics into several patterns. Unfortunately these subwords can still overlap in some position. This might lead to an overcount in the matching statistics, in which the same region of the string contributes more than once. Our aim is to remove the possibility of overcount by filtering the most representative common subwords for each region of the sequences *s*_1_ and *s*_2_, which will also remove all overlaps.

### Underlying subwords

When comparing entire genomes we want to avoid that large non-coding regions, which by nature tend to be highly repetitive, may contribute to the similarity score a multiple number of times, thus misleading the final score. In fact, while analyzing massive genomes, the number of repeated patterns is very high, particularly in the non-genic regions. Therefore we need to filter out part of this information, and select the “best” common subword, by some measure, for each region of the sequences.

In this regard, we must recall the definition of pattern priority and of underlying pattern, adapted from [26] to the case of pairwise sequence comparison. We will take as input the irredundant common subwords and the underlying quorum *u *= 2, i.e. they must appears at least twice. Let now *w* and *w*^*′*^ be two distinct subwords. We say that *w* has priority over *w*^*′*^, or *w*→*w*^*′*^, if and only if either |*w*| ≥ |*w*^*′*^|, or |*w*| = |*w*^*′*^| and the first occurrence of *w* appears before the first occurrence of *w*^*′*^. In this case, every subword can be defined just by its length and one of its starting positions in the sequences, meaning that any set of subwords is totally ordered with respect to the priority rule. We say that an occurrence *l* of *w* is *tied* to an occurrence *l*^*′*^ of a subword *w*^*′*^, if the two occurrences overlap, i.e. ([*l*, *l* + |*w*| − 1]∩*l*^*′*^, *l*^*′*^ + |*w*^*′*^| − 1]) ≠* ∅*, and *w*^*′*^→*w*. Otherwise, we say that *l* is *untied* from *l*^*′*^.

#### Definition 2

(Underlying subword) A set of subwords $U_{s_{1},s_{2}}\subseteq I_{s_{1},s_{2}}$ is said to be *underlying* if and only if: 

(i) every subword *w* in $U_{s_{1},s_{2}}$, called an *underlying subword*, has at least two occurrences, one in *s*_1_ and the other in *s*_2_, that are untied from all the untied occurrences of other subwords in $U_{s_{1},s_{2}}\setminus w$, and

(ii) there does not exist a subword $w\in I_{s_{1},s_{2}}\setminus U_{s_{1},s_{2}}$ such that *w* has at least two untied occurrences, one per sequence, from all the untied occurrences of subwords in $U_{s_{1},s_{2}}$.

This subset of $I_{s_{1},s_{2}}$ is composed only by those subwords that rank higher with our priority rule with respect to *s*_1_. In fact, if there are overlaps between subwords that are in $I_{s_{1},s_{2}}$, we will select only the subwords with the highest priority. Similarly to the score *ACS*(*s*_1_*s*_2_), the set $U_{s_{1},s_{2}}$ is asymmetric and depends on the order of the two sequences; we will address this issue in Section “A distance-like measure based on underlying subwords”. As for the underlying patterns [26], one can show that the set of underlying subwords exists, and is unique. As a corollary we know that the untied occurrences of the underlying subwords can be mapped into the sequences *s*_1_ and *s*_2_ without overlaps. Moreover, by definition, the total length of the untied occurrences cannot exceed the length of the sequences. These two properties are crucial when building a similarity measure, because any similarity that is based on these subwords will count the contribution of a region of the sequence only once.

### Efficient computation of underlying subwords

To summarize we select the irredundant common subwords that best fit each region of *s*_1_ and *s*_2_, employing a technique that we call *Underlying Approach* or, in short, UA. This technique is based on a simple pipeline. We first select the irredundant common subwords and subsequently filter out the subwords that are not underlying. From a different perspective, we start from the smallest set of subwords that captures the matching statistics and remove the overlaps by applying our priority rule. In the following we show how to compute the irredundant common subwords and the matching statistics, and then we present an approach for the selection of the underlying subwords among these subwords. The general structure of the Underlying Approach (UA) is the following: 

• 1) Compute the set of the irredundant common subwords $I_{s_{1},s_{2}}$

• 2) Rank all subwords in $I_{s_{1},s_{2}}$ according to the priority and initialize U to an empty set.

• 3) Iteratively select a subwords *p* from $I_{s_{1},s_{2}}$ following the ranking.

• 4a) If *p* has at least two untied occurrences: add *p* to U and update the corresponding regions of Γ(see next) in which *p* occurs;

• 4b) otherwise, discard *p* and return to (3).

#### Discovery of the irredundant common subwords

In step (1) we construct the generalized suffix tree $T_{s_{1},s_{2}}$ of *s*_1_ and *s*_2_. We recall that an occurrence of a subword is (left)right-maximal if it cannot be covered from the (left)right by some other common subword. The first step consists in making a depth-first traversal of all nodes of $T_{s_{1},s_{2}}$, and coloring each internal node with the colors of its leaves (each color corresponds to an input sequence). In this traversal, for each leaf *i* of $T_{s_{1},s_{2}}$, we capture the lowest ancestor of *i* having both the colors *c*_1_ and *c*_2_, say the node *w*. Then, *w* is a common subword, and *i* is one of its right-maximal occurrences (in *s*_1_ or in *s*_2_); we select all subwords having at least one right-maximal occurrence. The resulting set will be linear in the size of the sequences, that is *O*(*m* + *n*). This is only a superset of the irredundant common subwords, since the occurrences of these subwords could be not left-maximal.

In a second phase, we map the length of each right-maximal occurrence *i* into $l_{s_{1}}(i)$, and, using Proposition 1, we check which occurrences *i* have length greater than or equal to the length stored in the location *i*−1 (for locations *i *≥ 2). These occurrences are also left-maximal, since they cannot be covered by a subword appearing at position *i*−1. Finally we can retain all subwords that have at least an occurrence that is both right- and left-maximal, i.e, the set of irredundant common subwords $I_{s_{1},s_{2}}$. Note that, by employing the above technique, we are able to directly discover the irredundant common subwords and the matching statistics $l_{s_{1}}(i)$.

The construction of the generalized suffix tree $T_{s_{1},s_{2}}$ and the subsequent extraction of the irredundant common subwords $I_{s_{1},s_{2}}$ can be completed in time and space linear in the size of sequences.

#### Selection of the underlying subwords

In this section we describe, given the set of the irredundant common subwords $I_{s_{1},s_{2}}$, how to filter out the subwords that are not underlying, obtaining the set of underlying subwords $U_{s_{1},s_{2}}$.

The extraction of underlying subwords takes as input the set $I_{s_{1},s_{2}}$ and the tree $T_{s_{1},s_{2}}$ from the previous section. First we need to sort all subwords in $I_{s_{1},s_{2}}$ according to the priority rule (step 2). Then, starting from the top subword, we analyze iteratively all subwords by checking their untied occurrences (step 3). If the subword passes a validity test we select it as underlying (step 4a), otherwise we move on with the next subword (step 4b). The two key steps of this algorithm are: sorting the subwords (step 2) and checking for their untied occurrences (step 4a).

Step 2 is implemented as follows. For all subwords we retrieve their lengths and first occurrences in *s*_1_ from the tree $T_{s_{1},s_{2}}$. Then each subword is characterized by its length and the first occurrence. Since these are integers in the range [0,*n*] we can apply radix sort [27], first by length and then by occurrence. This step can be done in linear time.

In order to implement step 4a we need to define the vector Γ of *n* booleans, representing the locations of *s*_1_. If Γ[*i*] is true, then the location *i* is covered by some untied occurrence. We also preprocess the input tree and add a link for all nodes *v* to the closest irredundant ancestor, say *prec*(*v*). This can be done by traversing the tree in preorder. During the visit of a the node *v* if it is not irredundant we transmit to the children *prec*(*v*) otherwise if *v* is irredundant we transmit *v*. This preprocess can be implemented is linear time and space.

For each subword *w* in $I_{s_{1},s_{2}}$ we consider the list $L_{w}$ of occurrences to be checked. All $L_{w}$ are initialized in the following way. Every leaf *v*, that represent a position *i*, send its value *i* to the location list of the closest irredundant ancestor using the link *prec*(*v*). Again this preprocess takes linear time and space since all positions appear in exactly one location list. We will updated these lists $L_{w}$ only with the occurrences to be checked, i.e. that are not covered by some underlying subword already discovered. We start analyzing the top subword *w* and for this case $L_{w}$ is composed by all the occurrences of *w*.

For each occurrence *i* of *w* we need to check only its first and last location in the vector Γ; i.e., we need to check the locations Γ[*i*] and Γ[*i* + |*w*|−1]. If one of these two values is set to true, then *i* is tied by some subword *w*^*′*^. Otherwise, if both the values are set to false, then *i* must be untied from all other subwords. Since all subwords already evaluated are not shorter than *w*, then they cannot cover some locations in Γ[*i*,*i* + |*w*|−1] without also covering Γ[*i*] or Γ[*i* + |*w*|−1]. Thus, if Γ[*i*] and Γ[*i* + |*w*|−1] are both set to false, we mark this occurrence *i* as untied for the subword *w* and update the vector Γaccordingly.

If Γ[*i*] is true we can completely discard the occurrence *i*, for the subword *w* and also for all its prefixes, that are represented by the ancestors of *w* in the tree $T_{s_{1},s_{2}}$. Thus the occurrence *i* will no longer be evaluated for any other subword.

If Γ[*i*] is false and Γ[*i* + |*w*|−1] is true, we need to further evaluate this occurrence for some ancestors of *w*. In this case, one can compute the longest prefix, *w*^*′*^, of *w* such that Γ[*i* + |*w*^*′*^|−1] is set to false and *w*^*′*^is an irredundant common subword. Then the occurrence *i* is inserted into the list $L_{w^{\prime }}$.

This step is performed by first computing the length *d *< |*w*| such that Γ[*i* + *d*−1] is false and Γ[*i* + *d*] is true, and then retrieving the corresponding prefix *w*^*′*^ of *w* in the tree that spells an irredundant common subword with length equal to or shorter than *d*. We can compute *d* by means of a *length table χ* in support (or in place) of the boolean vector Γ. For each untied occurrence *i* of *w*, *χ *stores the values [1,2,…,|*w*|] in the locations [*i*,*i* + 1,…,*i* + |*w*|−1], similarly to the proof of Proposition 1. Using this auxiliary table we can compute the value of *d* for the location under study *i* as *d *= |*w*|−*χ*[*i* + |*w*|−1].

Now, to select *w*^*′*^, the longest prefix of *w* with |*w*^*′*^| ≤* d*, we employ an algorithm proposed by Kopelowitz and Lewenstein [28] for solving the *weighted ancestor problem*, where weights correspond to the length of words spelled in the path from the root to each node, in case of a suffix tree. In the weighted ancestor problem one preprocesses a weighted tree to support fast predecessor queries on the path from a query node to the root. That is, with a linear preprocessing on a tree of height *n*, using the above algorithm it is possible to locate any ancestor node *w*^*′*^ that has a weight less than *d* in time *O*(loglog*n*). In our case, the maximum length for an irredundant subword is min{*m*, *n*}, thus we can find a suitable ancestor *w*^*′ *^of *w* in time *O*(loglogmin{*m*,*n*}), with *O*(*m* + *n*) preprocessing of the tree $T_{s_{1},s_{2}}$.

At the end of the process, if the subword *w* has at least one untied occurrence per sequence, then we mark *w* as underlying subword. Otherwise, all the occurrences of *w* that are not covered are sent to its ancestors, using the previous procedure.

To analyze the overall complexity we need to compute how many times the same location *i* is evaluated. Suppose, for example, that *i* belongs to $L_{w}$ of the subword *w*. The location *i* is evaluated again for some $\bar{w}$, and inserted into the list $L_{\bar{w}}$, only if Γ[*i*] is false and Γ[*i* + |*w*|−1] is true. Note that the locations not already covered are in the range [*i*,*i* + |*w*|−*d*−1], with *d *> 0. Then, the subword $\bar{w}$ is the longest prefix of *w* that is an irredundant common subword and that lives completely in the locations [*i*,*i* + |*w*|−*d*−1]; however $\bar{w}$ may not cover the entire interval. Now, the occurrence *i* will be evaluated again only if there exists another subword *w*^*′*^ that overlaps with $\bar{w}$, and that has a higher priority with respect to $\bar{w}$. The worst case is when *w*^*′*^ends exactly at position *i* + |*w*|−*d*−1 and overlaps with $\bar{w}$ by only one location. Since *w*^*′*^must be evaluated before $\bar{w}$, then $\left|w^{\prime }|\geq |\bar{w}\right|$. Thus the worst case is when the two subwords have about the same length. In this settings the length of the subword $\bar{w}$ can be at most (|*w*|−*d*)/2. We can iterate this argument at most *O*(log|*w*|) times for the same position *i*. Therefore any location can be evaluated at most *O*(logmin{*m*,*n*}) times. In conclusion, our approach requires *O*((*m* + *n*)logmin{*m*,*n*}loglogmin{*m*,*n*}) time and *O*(*m* + *n*) space to discover the set of all underlying subwords $U_{s_{1},s_{2}}$.

### Extension to inversions and complements

In this section we discuss the extension of the algorithmic structure discussed above to accommodate also inversion and complement matches.

A simple idea is to concatenate each sequence with its inverse and its complement, while keeping separate the occurrences coming from direct matches, inversions, and complements. In brief, we first define $\hat{x}$ as the concatenation of a string *x* with its inverse, followed by its complement, in this exact order. Then, we compute the irredundant common subwords, $I_{s_{1},\hat{s_{2}}}$, on the sequences *s*_1_and $\hat{s_{2}}$. We subsequently select the underlying subwords by ranking all the irredundant common subwords in the set $I_{s_{1},\hat{s_{2}}}$. Using the same algorithm described above we compute the set $U_{s_{1},\hat{s_{2}}}$, and then we map each subword occurrence to the reference sequences *s*_1_. This will include also inversions and complements of *s*_2_ that are shared by *s*_1_. In this way, we can store all the untied occurrences and consider all possible matches for each region of *s*_1_.

In this framework, we choose to take into account all these symmetries, and thus the experiments presented will use this extended approach. We will also measure the contribution of inversions and complements to our similarity measure.

### A distance-like measure based on underlying subwords

In the following we report the basic steps of our distance-like measure. Let us assume that we have computed $U_{s_{1},s_{2}}$, and the other specular set $U_{s_{2},s_{1}}$. For every subword $w\in U_{s_{1},s_{2}}$ we sum up the score $h_{w}^{s_{1}}\overset{\left|w\right|}{\underset{i=1}{\sum }}i=h_{w}^{s_{1}}|w|(|w|+1)/2$ in *UA*(*s*_1_*s*_2_), where $h_{w}^{s_{1}}$ is the number of its untied occurrences in *s*_1_, similarly to ACS [7]. Then, we average *UA*(*s*_1_, *s*_2_) over the length of the first sequence, *s*_1_, yielding

$$\text{UA}(s_{1},s_{2})=\frac{\underset{w\in U_{s_{1},s_{2}}}{\sum }h_{w}^{s_{1}}|w|(|w|+1)}{2n}.$$

This is a similarity score that is large when two sequences are similar, therefore we take its inverse.

Moreover, for a fixed sequence *s*_1_ this score can also grow with the length of *s*_2_, since the probability of having a match in *s*_1_increases with the length of *s*_2_. For this reason, we consider the measure log_4_(|*s*_2_|)/*UA*(*s*_1_,*s*_2_); we use a base-4 logarithm since DNA sequences have four bases. Another issue with the above formula is the fact that it is not equal to zero for *s*_1_ =* s*_2_; thus we subtract the correction term log_4_(|*s*_1_|)/*UA*(*s*_1_,*s*_1_), which ensures that this condition is always satisfied. Since $U_{s_{1},s_{1}}$ contains only one subword, the sequence *s*_1_ itself, which trivially has only one untied occurrence in *s*_1_, this yields to *UA*(*s*_1_,*s*_1_) = |*s*_1_|(|*s*_1_| + 1)/(2|*s*_1_|) = (|*s*_1_| + 1)/2. The following formulas accommodate all of these observations in a symmetrical distance-like measure *d*_*UA*_(*s*_1_,*s*_2_) between the sequences *s*_1_ and *s*_2_:

$$\bar{\text{UA}}(s_{1},s_{2})=\frac{{\text{log}}_{4}(|s_{2}|)}{\text{UA}(s_{1},s_{2})}-\frac{2{\text{log}}_{4}(|s_{1}|)}{\left(|s_{1}|+1\right)},$$

$$d_{\text{UA}}(s_{1},s_{2})=\frac{\bar{\text{UA}}(s_{1},s_{2})+\bar{\text{UA}}(s_{2},s_{1})}{2}.$$

We can easily see that the correction term rapidly converges to zero as |*s*_1_| increases. Moreover, we notice that *d*_*UA*_(*s*_1_,*s*_2_) grows as the two sequences *s*_1_ and *s*_2_ diverge. From now we will simply refer to the measure *d*_*UA*_(*s*_1_,*s*_2_) as the Underlying Approach measure, or *UA*.

## Results

### Genome datasets and reference taxonomies

We assess the effectiveness of the Underlying Approach on the estimation of whole-genome phylogenies of different organisms. We tested our distance function on three types of datasets: viruses, prokaryotes, and unicellular eukaryotes.

In the first dataset we selected 54 virus isolates of the 2009 human pandemic *Influenza A – subtype H1N1*, also called the “Swine Flu.” The Influenza A virion has eight segments of viral RNA with different functions. These RNAs are directly extracted from infected host cells, and synthesized into complementary DNA by reverse transcription reaction, where a specific gene amplification is performed for each segment [29]. We concatenate these segments according to their conventional order given by the literature [30]; this step, in general, does not affect the final phylogeny computed by our algorithm, and is used to sort subwords by location. The resulting sequences are very similar to each other, and have lengths in the order of 13,200 nucleotides each, accounting for a total of 714,402 b. To compute a reference taxonomic tree, we perform multiple sequence alignment using the ClustalW2 [31] tool^a^ as suggested by many scientific articles on the 2009 Swine Flu [29,30]. Then, we compute the tree using the *Dnaml* tool from the PHYLIP [32] software package,^b^ which implements the maximum likelihood method for aligned DNA sequences. In *Dnaml* we used the parameters suggested in [29,30], which consider empirical base frequencies, constant rate variation among sites (with no weights), a transition ratio of 2.0, and best tree search based on proper searching heuristics.

In the second dataset we selected 18 prokaryotic organisms among the species used in [7] for a DNA phylogenomic inference. We chose the species whose phylogenomic tree can be inferred by well-established methods in the literature (see Table 2). The organisms come from both the major prokaryotic domains: Archaea, 8 organisms in total, and Bacteria, 10 organisms in total. The sequences in question have lengths ranging from 0.6 Mbp to 8 Mbp, accounting for a total 48 Mbp. We compute their tree-of-life by using genes that code for the 16S RNA, the main RNA molecule inside the small ribosomal subunit characterizing prokaryotes and widely used to reconstruct their phylogeny; the considered sequences are called 16S rDNA. We can extract a multiple alignment of 16S rDNA sequences of the selected organisms from the Ribosomal Database Project [33];^c^ our experiments are based on the release 8.1. Next, we perform a maximum likelihood estimation on the aligned set of sequences, employing *Dnaml* from PHYLIP with standard parameters, in order to compute a reference tree based on the resulting estimation.

**Table 2:** **Benchmark for prokaryotes – *Archaea *&*Bacteria *domains**

**Accession No.**	**Domain**	**Organism**	**Size**
BA000002	*archaea*	aeropyrum pernix str. K1	1.7 Mbp
AE000782	*archaea*	archaeoglobus fulgidus str. DSM 4304	2.2 Mbp
AE009439	*archaea*	methanopyrus kandleri str. AV19	1.7 Mbp
AE010299	*archaea*	methanosarcina acetivorans str. C2A	5.8 Mbp
AE009441	*archaea*	pyrobaculum aerophilum str. IM2	2.3 Mbp
AL096836	*archaea*	pyrococcus abyssi	1.8 Mbp
AE009950	*archaea*	pyrococcus furiosus str. DSM 3638	1.9 Mbp
AE000520	*archaea*	treponema pallidum sp. pall. str. Nichols	1.2 Mbp
AE017225	*bacteria*	bacillus anthracis str. Sterne	5.3 Mbp
AL009126	*bacteria*	bacillus subtilis subsp. subtilis str. 168	4.3 Mbp
AE013218	*bacteria*	buchnera aphidicola str. Sg	651 kbp
AL111168	*bacteria*	campylobacter jejuni sp. jej. str. NCTC 11168	1.7 Mbp
AE002160	*bacteria*	chlamydia muridarum str. MoPn/Wiess-Nigg	1.1 Mbp
AM884176	*bacteria*	chlamydia trachomatis str. L2/434/Bu	1.1 Mbp
AE016828	*bacteria*	coxiella burnetii str. RSA 493	2.0 Mbp
AE017285	*bacteria*	desulfovibrio vulgaris sp. vulg. str. Hildenb.	3.6 Mbp
L42023	*bacteria*	haemophilus influenzae str. Rd KW20	1.9 Mbp
CP001037	*bacteria*	nostoc punctiforme str. PCC 73102	8.4 Mbp

Prokaryotic taxa used in our experiments, divided by domain. For each entity, we list the accession number in the NCBI genome database, the complete name and strain, and the genome size.

In the third dataset we selected five eukaryotic organisms of the protozoan genus *Plasmodium* whose genomes have been completely sequenced (Table 3). *Plasmodium* are unicellular eukaryotic parasites best known as the etiological agents of malaria infectious disease. The sequences have lengths ranging from 18 Mbp to 24 Mbp, accounting for a total 106 Mbp. We used as reference tree the taxonomy computed by Martinsen *et al.*[34], as suggested by the Tree of Life Project.

**Table 3:** Plasmodium are parasites known as causative agents of malaria in different hosts and geographic regions

**Parasite**	**Host**	**Region**	**Size**
P. berghei	rodent	Africa	18.5 Mbp
P. chabaudi	rodent	Africa	18.8 Mbp
P. falciparum	human	Africa, Asia & S./C. America	23.3 Mbp
P. knowlesi	macaque	Southeast Asia	23.7 Mbp
P. vivax	human	Africa, Asia & S./C. America	22.6 Mbp

The right-most column lists the size of each complete DNA genome.

### Whole-genome phylogeny reconstruction

We exploited the above datasets to compare our method, the Underlying Approach (UA), with other efficient state-of-the-art approaches in the whole-genome phylogeny reconstruction challenge: ACS [7], FFP [4]^d^ and FFP_*RY*_. The FFP_*RY *_method, in contrast to FFP, employs the Purine-Pyrimidine reduced alphabet (RY) which is composed by two character classes: [A,G] (both purine bases, denoted by R) [C,T] (both pyrimidines, denoted by Y). We implemented the ACS method by ourselves, while for FFP and FFP_*RY*_ we used the FFP package release 3.14 available online.

We reconstruct the phylogenomic trees from the distance matrices using the Neighbor-joining method as implemented in the PHYLIP package. We compare the resulting topologies with the respective reference trees using the symmetric difference of Robinson and Foulds (R-F) and the triplet distance. For two unrooted binary trees with *n *≥ 3 leaves, the R-F score is in the range [0,2*n*−6]. A score equal to 0 means that the two trees are isomorphic, while 2*n*−6 means that all non-trivial bipartitions are different. The R-F difference between two or more trees can be computed using the TreeDist tool from the PHYLIP package.

We ran FFP and FFP_*RY *_for different values of *k* (the fixed subword length) as suggested by [4], retaining the best results in agreement with the reference trees. Table 4 compares our method with the other state-of-the-art approaches, by showing the R-F difference with respect to the reference taxonomy tree.

**Table 4:** Comparison of whole-genome phylogeny reconstructions

**Species**	**Group**	**UA**	**ACS**	**FFP**	**FFP**_***RY***_
Influenza A	Viruses	**80**/102	84/102	100/102	96/102
*Archaea*	Prokaryotes	**4**/10	**4**/10	6/10	6/10
*Bacteria*	Prokaryotes	**6**/14	10/14	**6**/14	10/14
Arch. & Bact.	Prokaryotes	**20**/30	22/30	**20**/30	22/30
Plasmodium	Eukaryotes	**0**/4	**0**/4	4/4	**0**/4

Normalized Robinson-Foulds scores with the corresponding reference tree. For each dataset the best results are in bold.

**Figure 1:**
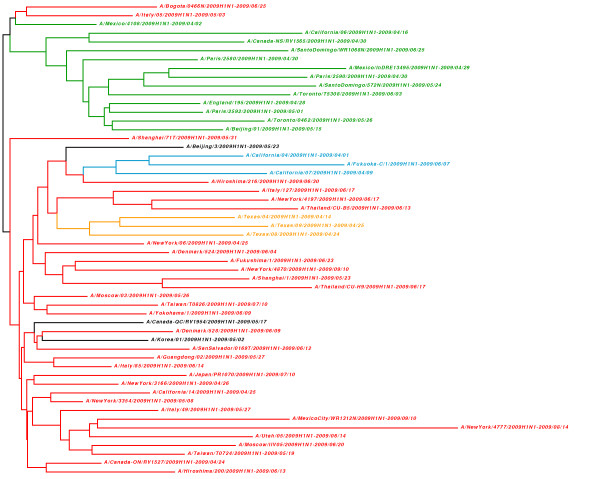
**Whole-genome phylogeny of the 2009 world pandemic Influenza A (H1N1) generated by UA.** In green and red are represent the two main clades, where the green Mexico/4108 is probably the closest isolate to the origin of the influenza. In blue and orange are two of the possible early evolutions of the viral disease. The organisms which do not fall into one of the two main clades according to the literature are in black.

**Figure 2:**
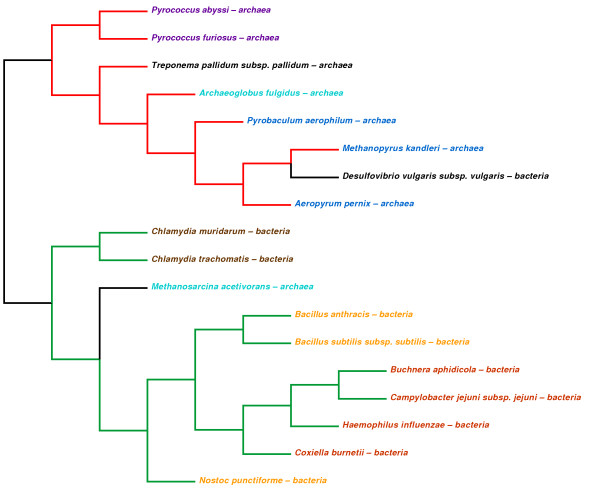
**Whole-genome phylogeny of prokaryotes by UA.** In red are the branches of the *Archaea* domain, while in green are those of the *Bacteria* domain. Clusters of other organisms are highlighted with different colors. Only two organisms do not fall into the correct clade: Methanosarcina acetivorans – archaea (in cyan) and Desulfovibrio vulgaris subspecies vulgaris – bacteria (in black).

**Figure 3:**
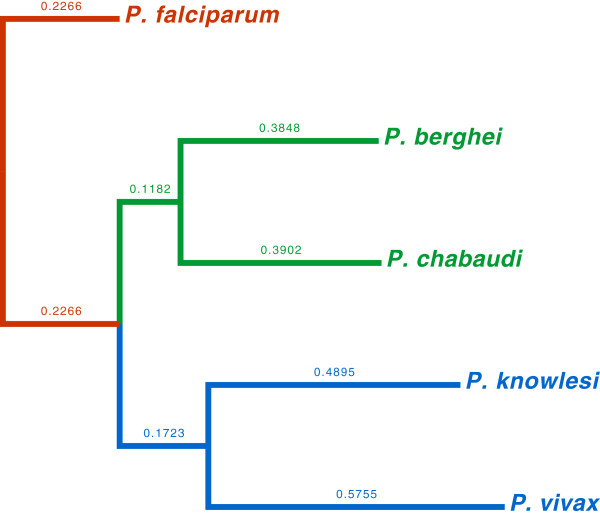
**Whole-genome phylogeny of the genus *Plasmodium *by UA, with our whole-genome distance highlighted on the branches.**

Our method, UA, achieves good performance in every test considering the R-F difference with the reference taxonomy tree, and very good performance if we further analyze the resulting phylogenies, as in Figures 1, 2, and 3. For every dataset the best results are shown in bold. We can observe that UA is constantly the best performing method, and that this advantage becomes more evident for large dataset, where sequences share large parts, such as the Influenza A (H1N1) viruses.

The Robinson and Foulds distance is a standard method to evaluate topological discordance between trees. However when dealing with large trees it is known that small variations can generate very large R-F scores (typically, already for *n*∼10). For this reason we conducted a second series of experiments using the triplet distance [35]. The triplet distance is a more refined measure that does not suffer this problem. Moreover, to better compare all taxonomies, we report the triplet distance between all trees. Tables 5, 6 and 7 show the triplet distance between all trees for all datasets. This more refined measure confirms the applicability of UA with respect to FFP and ACS.

**Table 5:** Comparison of whole-genome phylogeny of influenza virus

**Viruses**	**Reference**	**UA**	**ACS**	**FFP**	**FFP**_***RY***_
Reference	0.0	0.60	0.63	0.86	0.88
UA	**0.60**	0.0	0.30	0.81	0.74
ACS	0.63	0.30	0.0	0.83	0.81
FFP	0.86	0.81	0.83	0.0	0.73
FFP_*RY*_	0.88	0.74	0.81	0.73	0.0

Normalized triplet distance between all trees. The best results are in bold.

**Table 6:** Comparison of whole-genome phylogeny of prokaryotes

**Prokaryotes**	**Reference**	**UA**	**ACS**	**FFP**	**FFP**_***RY***_
Reference	0.0	0.24	0.37	0.62	0.39
UA	**0.24**	0.0	0.37	0.55	0.47
ACS	0.37	0.37	0.0	0.59	0.48
FFP	0.62	0.55	0.59	0.0	0.57
FFP_*RY*_	0.39	0.47	0.48	0.57	0.0

Comparison of Whole-Genome Phylogeny of Prokaryotes. Normalized triplet distance between all trees. The best results are in bold.

**Table 7:** **Comparison of whole-genome phylogeny of *Plasmodium***

**Plasmodium**	**Reference**	**UA**	**ACS**	**FFP**	**FFP**_***RY***_
Reference	0.0	0.0	0.0	0.4	0.0
UA	**0.0**	0.0	0.0	0.3	0.0
ACS	**0.0**	0.0	0.0	0.3	0.0
FFP	0.4	0.3	0.0	0.0	0.3
FFP_*RY*_	**0.0**	0.0	0.0	0.3	0.0

Normalized triplet distance between all trees. The best results are in bold.

In more detail, Figure 1 shows that our approach can distinguish the two main clades of the 2009 Influenza A-H1N1 (in green and red), which have been outlined in [30]. The origin of the flu could reside in the Mexican isolate (Mexico/4108, in green), from which all other green isolates may have ensued. Two sub-clades for the U.S. states of California and Texas are highlighted within the red clade, most probably corresponding to the first major evolutions of the viral disease.

Similar results are obtained for the second dataset, as shown in Figure 2. UA can easily distinguish the Archaea domain, in red, from the Bacteria domain, in green, and also other sub-clades with respect to the reference tree (these sub-clades are highlighted in the figure with different colors). The organisms in black do not form a clade with other organisms in the reference tree. For the third dataset (Figure 3), the whole-genome phylogeny of the genus *Plasmodium* generated by UA corresponds exactly to the taxonomy found in the literature.

The accuracy results are promising, but we believe that of equal interest are the patterns used for the classification. Our approach, by construction, uses only a very small number of patterns. For this reason we report in Table 8 some statistics for the underlying subwords selected, averaged over all experiments. We can notice that the number of irredundant patterns is in general smaller than the length of the genomes, and this is a first form of information filtering. Moreover we can observe that only a few underlying subwords are selected on average among the irredundant common subwords. This number is always very small when compared with all possible irredundant subwords, and much smaller than the length of the sequences.

**Table 8:** Main statistics for the underlying approach averaged over all experiments

**Counting**	**Influenza A**	**Arch. & Bact.**	**Plasmodium**
Min genome size	12,976 b	650 kbp	18,524 kbp
Max genome size	13,611 b	8,350 kbp	23,730 kbp
Average genome size	13,230 b	2,700 kbp	21,380 kbp
Irredundants $\left|I_{s_{1},s_{2}}\right|$	3,722	3,167 k	16,354 k
Underlying subwords	60	112 k	706 k
$\left|U_{s_{1},s_{2}}\right|$			
Min |*w*| in $U_{s_{1},s_{2}}$	6	10	12
Max |*w*| in $U_{s_{1},s_{2}}$	1,615	25	266
Average |*w*| in $U_{s_{1},s_{2}}$	264	14	20
Untied inversions	28%	31%	33%
Untied complements	22%	20%	19%

Similar considerations can be drawn for the underlying subwords length. On average they can be very long, especially with respect to FFP that uses only k-mers with *k* in the range [5,10]. Furthermore, each underlying subword occurs only a few times per sequence, and in general about one occurrence per sequence. Removing the high-frequency subwords, we can notice that the underlying subwords typically have length ≥ log_4_ min{*m*,*n*}, and in the case of viruses they can be very large, capturing more information than FFP. The longest underlying subwords appear in the virus dataset, and they are on the order of a thousand bases. We checked if these subwords may have some biological meaning and we found that in some cases they correspond to whole viral segments that are shared between two genomes. This confirms that, in some cases, the underlying subwords used for classification can capture some biological insight.

Another interesting aspect is the contribution of inversions and complements in our similarity measure, with respect to the classical notion of match. We compute the average number of occurrences used in our scoring function that is due to inversions and complements. The contribution of inversions and complements is about 28-33% and 19-20%, respectively. This fact may be due to the nature of the sequences considered, but we believe that this topic deserves more attention.

## Conclusion

In conclusion, we have shown that the underlying subwords can be used for the reconstruction of phylogenetic trees. Preliminary experiments have shown very good performance in the identification of major clusters for viruses, prokaryotes, and unicellular eukaryotes. An important observation that distinguishes our methods from the others is that only a small number of underlying subwords is used in our distance, nevertheless the results are promising. From this fact we can speculate that only a very limited number of subwords is needed to establish the phylogeny of genomic sequences. Thus, an interesting problem that can be addressed using the underlying subwords is the selection of probes for DNA chips.

In the future, we plan to extend this method for the comparison of whole genomes based on short reads coming from next-generation sequencing, instead of using assembled genomes.

## Endnotes

^a^ ClustalW2 is available at http://www.ebi.ac.uk/Tools/msa/clustalw2.

^b^ PHYLIP (phylogenetic inference package) is a free computational phylogenetics software package available at http://evolution.genetics.washington.edu/phylip.

^c^ The Ribosomal Database Project is available at http://rdp.cme.msu.edu.

^d^ The FFP software package release 3.14 is available at http://ffp-phylogeny.sourceforge.net.

## Competing interests

The authors declare that they have no competing interests.

## Authors’ contributions

All authors contributed equally to this study. All authors read and approved the final manuscript.
